# Candida auris: a novel emerging nosocomial pathogen – properties, epidemiological situation and infection control 

**DOI:** 10.3205/dgkh000444

**Published:** 2023-08-16

**Authors:** Marcelo Caliman Sato, Emilene Cristine Izu Nakamura Pietro, Lucas Marques da Costa Alves, Axel Kramer, Paulo Sérgio da Silva Santos

**Affiliations:** 1 Center for Lasers and Applications, Instituto de Pesquisas Energéticas e Nucleares (IPEN-CNEN), São Paulo, Brazil; 2 Bauru State Hospital, Bauru, São Paulo, Brazil; 3 Institute of Hygiene and Environmental Medicine, University Medicine Greifswald, Greifswald, Germany; 4 Bauru School of Dentistry of University of São Paulo, Bauru, São Paulo, Brazil

## Abstract

Immunosuppression and critical illnesses in combination with ecological imbalance open the door for novel opportunistic fungal infections, as in case of *Candida (C). auris*. *C. auris* has emerged globally as a multidrug-resistant yeast, causing infections and outbreaks in health care facilities. This narrative review discusses the properties of the yeast, the development of the epidemiological situation, the nosocomial spread and causes for nosocomial outbreaks triggered by *C. auris* in the hospital environment, and summarizes international recommendations for infection control, supplemented by suggestions on diagnostic, screening and antibiotic stewardship.

## Abstract (de)

Immunsuppression und kritische Erkrankungen öffnen in Kombination mit einem ökologischen Ungleichgewicht die Tür für neuartige Infektionen durch opportunistische Pilze wie im Fall von *Candida (C). auris*. *C. auris*, einem multiresistentem Hefepilz. *C. auris* hat sich weltweit ausgebreitet und kann Infektionen und Ausbrüche in Gesundheitseinrichtungen verursachen. In dem narrativen Review werden die Eigenschaften des Erregers, die Entwicklung der epidemiologischen Situation, die nosokomiale Ausbreitung und die Ursachen für nosokomiale Ausbrüche, die durch *C. auris* im Krankenhausumfeld getriggert werden, erörtert, internationale Empfehlungen zur Infektionskontrolle zusammengefasst und ergänzende Hinweise zu Diagnostik, Screening und Antibiotic Stewardship gegeben.

## Epidemiologic development

*Candida (C.) auris* was first described as causative agent of otomycosis in Japan in 2009 [1]. The clonal spread of *C. auris* has now reached Middle East, Africa, South America, North America and Europe, posing a severe global health hazard [2]. In the period from 2013 to 2017, 620 detections of *C. auris* were reported in Europe, of which 24.8% involved infection [3]. In Germany, only a few (mostly introduced) cases have been reported so far [4]. The Pan American Health Organization/World Health Organization (PAHO/WHO) issued an epidemiological alert in October 2016 in response to reports of *C. auris* outbreaks in Latin American healthcare facilities [5]. The high rate of transmissibility and mortality, which ranged from 30 to 60% of persons afflicted, prompted the alert. The first major epidemic in Latin America occurred in Venezuela in 2013 at an intensive care unit of a tertiary care hospital in Maracaibo. The outbreak affected 18 individuals, 13 of whom were children, with a 28% case fatality rate [5]. Three years later, in 2015, the world’s worst outbreak occurred in London, affecting 50 patients in one hospital; 22 were infected and 28 were colonized [6]. Resulting a systematic study and global meta-analysis covering a decade, more than 4,000 cases of *C. auris* were reported from at least 33 countries with high resistance to fluconazole, moderate resistance to amphotericin B and caspofungin, high sensitivity to micafungin and anidulafungin, and a mortality rate of 45% in cases of disseminated infections [7]. According to a 2020 study, *C. auris* is now established in 43 countries across five continents (Australia, Bangladesh, Canada, China, Colombia, France, Germany, India, Israel, Japan, Kenya, Kuwait, Malaysia, Netherlands, Oman, Pakistan, Panama, Qatar, Russia, Saudi Arabia, Singapore, South Africa, South Korea, Spain, Sudan, Switzerland, United Kingdom, United States, Venezuela, Austria, Belgium, Chile, Costa Rica, Egypt, Greece, Italy, Iran, Mexico, Norway, Poland, Taiwan, Thailand, United Arab Emirates). In 2022 were 2,377 clinical cases and 5,754 screening cases [8]. 

*C. auris* is an example of the consequences of uncontrolled ecology increased by the Coronavirus pandemic. With the rise in COVID-19 infections, a trend of bacterial, fungal, and viral superinfection has been noted. Due to the multidrug-resistance and easy transmissibility, *C. auris* is difficult to manage in COVID-positive patients [9]. The first case of *C. auris* from Brazil (strain I) was published in 2021 [10]. Despite the fact that the microorganism was isolated from a catheter from an adult patient in an intensiv care unit (ICU) in Salvador, Bahia state, Brazil, due to COVID-19 complications, the first confirmation of this microorganism occurred only on December 07, 2020, despite the fact that the patient was not contaminated [10]. 

## Characteristics of C. auris

### Phylogenetic analysis

Initial phylogenetic analysis of *C. auris* revealed four populations named after the geographical location South Asia, East Asia, Africa, and South America (clade I, II, III and IV, respectively) [11], [12], that corresponded to these geographical regions [5]. Most strains showed phylogeographic mixing; strain IV with isolates primarily from South America showed the strongest phylogeography [5]. In the meantime, whole genome sequencing (WGS) analysis confirmed the existence of a fifth clade by providing WGS data of another four Iranian isolates [13]. 

### Pathopotency

*C. auris* was named after the first case of otomycosis and is derived from the Latin word for ear (auris). However, despite the name, *C. auris* colonizes asymptomatically on mucous membranes (nose, throat), is detectable on the skin (axilla, ear, inguinal area), in wounds, respiratory samples and stool as well as urine, and is a pathogen of invasive infections such as that of the bloodstream [5] and urinary tract [14]. Asymptomatic colonization represents a risk for *C. auris* transmission. The future significance for oropharyngeal infections cannot yet be assessed [15]. 

The ANVISA Technical Standard (Agência Nacional de Vigilância Sanitária – Brazil) suggests that the risk factors for *C. auris* infections are similar to those for infections caused by other Candida species. Diabetes mellitus, the presence of a central venous catheter, immunosuppression, neutropenia, broad-spectrum antibiotic exposure, parenteral nutrition, blood transfusion, hemodialysis, surgery within 30 days, admission to an intensive care unit, antifungal agents received within 30 days, concomitant bacteremia, concomitant candidemia, indwelling urinary catheter, candiduria, chronic kidney disease, and recent chemotherapy are among the 12 risk factors known to date. Patients of all ages, from premature infants to the elderly, have been diagnosed with infections [16]. More research may be needed to learn more about *C. auris* infection, including early detection, risk factors, and treatments.

Neutrophils play an important role in the control of invasive fungal infections such as candidiasis. They combat yeasts by phagocytosis or by producing neutrophil extracellular traps (NETs) [17]. These neutrophils have web-like chromosomal DNA structures decorated with histones and antimicrobial effector molecules such as proteins. Human neutrophils do not recognize *C. auris*, indicating an innate weakening of the immune response to this yeast [17].

According to a study conducted in UK, healthcare spending related to an outbreak of this infection increased by about 10%, owing primarily to prolonged hospitalization time in infected individuals. Expenditures for complementary examinations were also high and persisted long after the infectious outbreak ended [18].

### Resistance

More than 90% of *C. auris* isolates from clade I and III and about half of the isolates from clade IV are resistant to fluconazole [12], [19], [20]. In addition, antifungal resistance against polyenes and echinocandins has been reported in these clades. Typically, clade II isolates are susceptible to azoles and other common antifungals [21], [22]. The detection of multi- or even pan-resistant *C. auris* strains is alarming [23].

### Laboratory characteristic

The morphology of *C. auris* may resemble other, more common, *Candida* spp.; thus, identification based only on colonies is not possible [24]. Most commercial biochemical tests commonly used may misidentify *C. auris* [25].

*C. auris* is a para-extended ovoid budding yeast that seldom produces pseudohyphae [26]. This organism can withstand high levels of salinity and heat. *C. auris* may be distinguished from other *Candida* species by its ability to grow at temperatures as high as 42°C [26]. In culture, some strains of *C. auris* have been shown to form aggregations, which may help the organism resist detergents, ultraviolet radiation, and antimicrobial agents. *C. auris* creates biofilms, which act as a surface adhesion mechanism [26]. However, due to the rarity of pseudohyphae, these biofilms are substantially thinner and less complex than those of *Candida albicans* [26].

Mistaken identification as other yeasts may occur when using standard biochemical methods and commercially available tests. Correct identification at the species level requires more advanced techniques, i.e., DNA sequencing or matrix-assisted laser desorption/ionization time-of-flight (MALDI-TOF), or both [27]. When invasive non-albicans Candida species are found, infection rates are on the rise, or after patients are admitted from a facility reporting a *C. auris* epidemic, and if the local laboratory lacks any specialized diagnostic techniques, the only option is clarification by a reference laboratory, where the isolates also are tested for antifungal susceptibility. According to a recent study of the diagnostic capacity of mycology laboratories in Latin America, only 16.6% of the centers in Brazil had this structure, while 15.5% of the centers could perform fungal DNA sequencing (9.5% could do both) [28].

## Infection control

### Necessity

The importance of controlling this opportunistic pathogen is due to

- high transmissibility [29], [30] and tenacity for 4–14 days [31], [32], [33]
- resistance to several antifungal drugs
- risk of causing bloodstream and other disseminated infections
- propensity to cause outbreaks due to difficult identification
- endangerment of patients with immunosuppression or immunodeficiency, as in the case of Covid-19, HIV or after transplantation.

Prompt notification is essential to implement infection control precautions in a timely manner and to ensure vigilance for development of infections in patients found to be colonized [34]. The detection of a case of *C. auris* should trigger an investigation including a detailed case review and screening of close-contact patients for *C. auris* carriage [25], because *C. auris* is distinguished from other *Candida* species by its transmissibility and high level of resistance to antifungal medications. 

In Germany, it is recommended to send isolated *C. auris* strains to the National Reference Center for Invasive Fungal Infections in order to record the epidemiological development [30].

### Transmission

*C. auris* is mainly transmitted through contact (hands, medical devices, near-patient environment including walls and floor). The environment may be the principal reservoir of* C. auris*, with transmission occurring through contaminated surfaces and equipment, such as patient-care equipment (stethoscopes, thermometers, etc.) or direct contact with persons [30]. Despite infection control measures, the yeast persists and spreads, indicating a resistance to environmental conditions, high transmissibility, and the ability to quickly colonize the patient’s skin and the surrounding environment [30]. After treating the infection, patients may remain colonized for up to 3 months [30].

### Infection control measures

Contact precaution, single room or cohorting of colonized or infected patients as well as pre-emptive isolation of contact patients or enhanced barrier precautions are recommended (Table 1 (Tab. 1)). As there are currently no established protocols for decolonization and determining when it is safe to end isolation, these precautions need to be applied until discharge from the hospital. Screening (see above) of close contacts of identified cases for *C. auris* carriage with axilla and groin swabs is important. Other sites (urine, wounds, catheter exit sites, throat etc.) can be sampled, if clinically relevant or indicated.

Hand antisepsis with alcohol-based hand rubs is essential to interrupt cross infections (Table 1 (Tab. 1)). Chlorhexidine gluconate (CHG) has shown some efficacy *in vitro*, but there are reports of patients with persistent colonization despite a twice-daily antiseptic body wash with CHG-based products [35]. Alcohol-based hand rubs are the preferred hand hygiene method for *C. auris*. If hands are visibly soiled, first wash with soap and water [36], dry thoroughly (because wet skin decreases the efficacy of alcohol-based hand rubs) [37], and thereafter perform hand antisepsis. The combination of washing and hand antisepsis can be expected to be effective, since in model tests on porcine skin a log reduction of 2.8 was achieved by washing for 30 s and a 3-log reduction using a 30-s rub-in of ethanol-based handgel (75% v/v) [38]. Wearing gloves is not a substitute for hand hygiene [36].

During isolation, daily disinfecting cleaning of the floor and near-patient surfaces is required. After discharge of patients, terminal disinfecting cleaning is necessary [39]. 

Depending of the formulation of the surface disinfectant, QAV may be less effective. The CDC and the ECDC therefore recommend sporocidal surface disinfectants [34], [36] such as oxidants, peracetic acid or chlorine-based disinfectants (i.e. 1,000 ppm sodium hypochlorite). After patient discharge, room disinfection by vaporization with hydrogen peroxide is an effective alternative to traditional wiping. 

Analogously, disinfection of reusable equipment (e.g., monitoring devices, thermometers, pulse oximeters, blood pressure measuring instruments, etc.) is performed with sporocidal disinfectans. 

Environmental sampling or screening of healthcare workers are not routinely recommended [36].

## Prevention of spreading from the hospital

In the USA, the detection of *C. auris* is manditorially nationally notifiable since 2018 [40]. In Germany, it is legally required that the receiving facility or the general practitioner be informed in the transfer sheet *(Überleitungsbogen*) on colonization or previous infection with a multidrug-resistant organism (MDRO) or other critical pathogens such as *C. auris* [41].

## Antimicrobial stewardship (ABS)

Although there is no evidence for the influence of ABS on the emergence and spread of *C. auris*, it is likely that an environment with a high level of broad-spectrum antibacterial and antifungal use will select multidrug-resistant yeasts. Therefore, antifungal prophylaxis should be weighed carefully in settings with evidence of *C. auris* transmission [34]. Additionally, colonization is not an indication for antifungal treatment, because it does not eradicate *C. auris* [16].

Health surveillance tools are needed to prevent the spread of new diseases globally [42].

Immunosuppressive drugs used to treat COVID-19 cases open the door for fungal infections, for instance, with *C. auris*, which, in combination with the ecological imbalance, is causing novel illnesses in humans (e.g., opportunistic fungal infection) [43].

The emergence of antifungal resistance poses another threat to human health and food safety, necessitating the development of novel antifungals [42]. The β-glucan synthesis inhibitor SCY-078 and rezafungin (previously named CD101) show promise as therapeutic possibilities. Turbinmicin, a chemical generated by a marine microbiome, named after the species from which it was isolated, and *Ecteinascidia corneto*, are intriguing therapy options. *Candida auris* and *Aspergillus fumigatus* were eliminated by this chemical with no negative effects in *in vitro* and mouse trials [44].

## Concluding remarks

The widespread use of antifungal drugs, especially of fluconazole (resistance in *C. auris* in >70%) in ICU patients, deficiencies in standard precautions and delayed diagnosis are the main reasons for outbreaks [2]. Patients with severe immunosuppression, induced by drugs or illness, and critically ill patients are particularly at risk. In combination with rising ambient temperatures, which might have selected thermotolerant yeasts in wetlands, travelling and spreading through different ecosystems and hosts (e.g., birds), has resulted in an ecological imbalance. Tolerance to high salinity levels and the ability to grow at 42°C argue for the latter hypothesis [45], [46], [47]. Presumably, the interaction of all these factors paves the way for novel illnesses caused by opportunistic fungal infections, such as *C. auris* [43]. 

Health surveillance tools are needed to prevent the spread of new diseases globally [42]. The analysis of outbreaks in different countries necessitates the development of an efficient strategy to restrict the spread of *C. auris* in Brazil and other nations that lack the essential infrastructure [16]. Detection of even a single case of *C. auris* should trigger an epidemiological investigation of an outbreak [34] and the immediate start of infection control measures to prevent further spread.

## Notes

### Competing interests

The authors declare that they have no competing interests.

### Authors’ ORCID:

- Marcelo Caliman Sato: 0009-0006-2964-2690
- Emilene Cristine Izu Nakamura Pietro:
0000-0002-4113-3980
- Lucas Marques da Costa Alves:
0000-0001-9018-6395
- Axel Kramer: 0000-0003-4193-2149
- Paulo Sérgio da Silva Santos: 0000-0002-0674-3759

## Figures and Tables

**Table 1:**
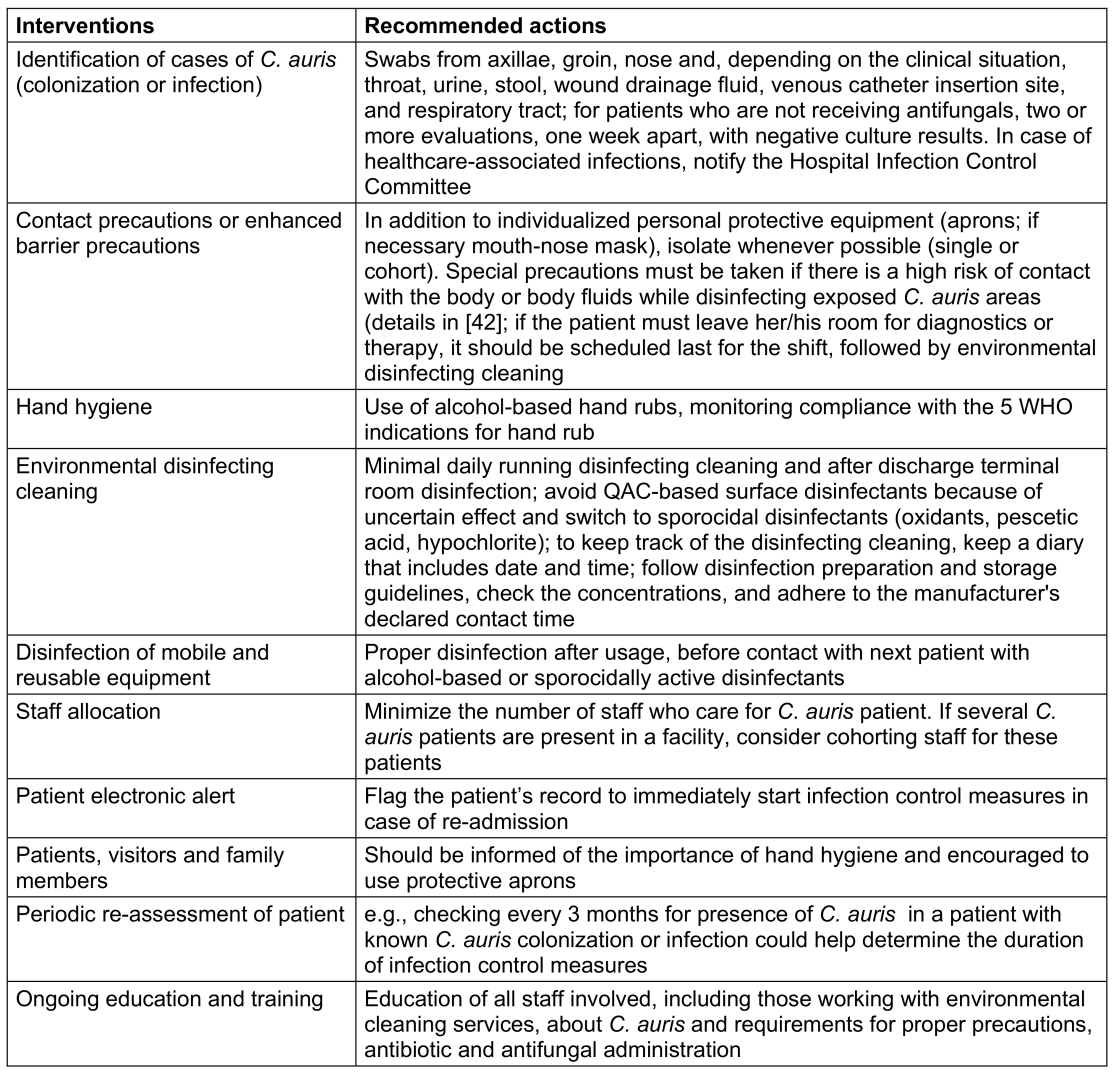
Summarized recommendations from infection control societies [7, 29, 36, 39, 40, 41] for infection control of *C. auris*
